# Peste des Petits Ruminants Virus, Mauritania

**DOI:** 10.3201/eid2002.131345

**Published:** 2014-02

**Authors:** Ahmed Salem El Arbi, Ahmed Bezeid El Mamy, Habib Salami, Ekatarina Isselmou, Olivier Kwiatek, Geneviève Libeau, Yaghouba Kane, Renaud Lancelot

**Affiliations:** Ministère du Développement Rural, Nouakchott, Mauritania (A.S. El Arbi);; Centre National d’Elevage et de Recherche Vétérinaire, Nouakchott (A.B. El Mamy, E. Isselmou);; Centre de coopération internationale en recherche agronomique pour le développement, Montpellier, France (H. Salami, O. Kwiatek, G. Libeau, R. Lancelot);; Institut National de la Recherche Agronomique, Montpellier (H. Salami, O. Kwiatek, G. Libeau, R. Lancelot);; Ecole Inter-Etats des Sciences et Médecine Vétérinaires de Dakar, Dakar, Senegal (Y. Kane)

**Keywords:** peste des petits ruminants virus, PPRV, peste des petits ruminants, PPV, viruses, Mauritania, sheep, goats, ruminants

**To the Editor:** Peste des petits ruminants virus (PPRV; genus *Morbillivirus*, family *Paramyxoviridae*) causes severe infectious disease in sheep and goats in Africa and Asia. Pneumo-enteritis clinical signs are dominated by ocular and nasal discharge, and mortality rates are high (*1*). Four distinct lineages of PPRV have been described on the basis of a phylogenetic analysis of a cDNA fragment of the nucleoprotein (NP) gene (*2*): lineages I and II are found in western Africa (*1*,*3*,*4*), lineage III in eastern Africa and the Middle East, and lineage IV in Asia. Recent studies have shown changes in this distribution (*1*,*5*), including the emergence of PPRV lineage IV in northeastern and northern Africa (*5*). Sparse serologic results (*6*,*7*) are available regarding PPRV spread in Mauritania or genetic features of circulating PPRV strains.

A seroprevalence survey was implemented in October 2010 to assess PPRV spatial distribution in Mauritania. The study was limited to 8 southern provinces (*wilayas*), which covered 99.3% of the national sheep and goat stocks (Technical Appendix Figure 1). Of 40 districts (*mougataas*), 21 were randomly selected. A single geographic point was randomly sampled within each of the selected *mougataas*, and 100 small ruminants were sampled in a 7-km radius around the coordinates. None of the *mougataas* in which sampling occurred had a PPRV vaccination program.

In addition, field veterinary officers from Trarza and Tagant Provinces were asked to report suspected outbreaks of peste des petits ruminants disease (PPR) during January–March 2012. All outbreaks were investigated, and biologic samples were collected for laboratory diagnostics. 

All serum samples from the 2010 and 2012 surveys were analyzed by using antibody ELISA ID Screen PPR competition (IDvet Innovative Diagnostics, Grabels, France). Optical density values were converted to inhibition percentages; according to the ELISA cutoff value, inhibition percentages of <45% were considered positive. A logistic beta-binomial regression model was used to analyze prevalence rates within mougataas. Swab samples were tested by using reverse transcription PCR (RT-PCR) adapted to a 1-step format (OneStep RT-PCR Kit; QIAGEN, Hilden, Germany) and based on nucleoprotein (NP) 3–NP4 PPRV-specific primers targeting the 3′ end of the NP gene (*8*). Amplicons of 351 nt were extracted, and after sequencing, nucleic acid segments were aligned with PPRV sequences stored in the database of the Centre de coopération internationale en recherche agronomique pour le développement (Montpellier, France) or retrieved from GenBank (Figure).

**Figure:**
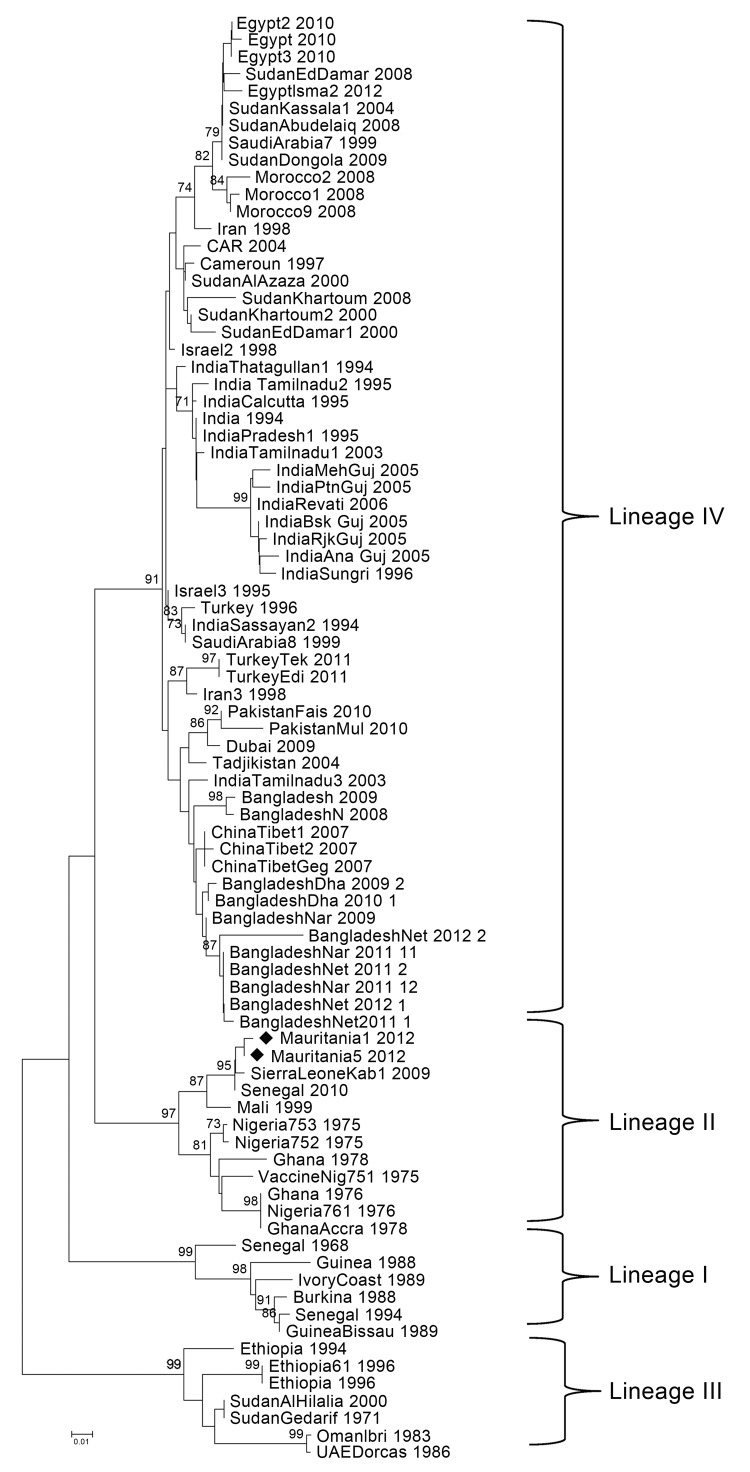
Phylogenetic tree based on the nucleoprotein gene of peste des petits ruminants viruses identified in Mauritania (black diamonds) and selected comparison sequences from GenBank. The neighbor-joining method was used for phylogenetic analysis; evolutionary distances were computed by using the Tamura 3-parameter method and a gamma distribution parameter with a value of 4 (*9*). CAR, Central African Republic; Nig, Nigeria; UAE, United Arab Emirates. Scale bar indicates nucleotide substitutions per site.

A total of 1,190 sheep and 714 goat serum samples were collected during the 2010 survey; the estimated serologic PPRV prevalence rate was 43% (n = 1,904; 95% CI 38%–47%). PPRV infection was widespread: prevalence rates ranged from 3% (Guerou) to 98% (Kobeni) (Technical Appendix Figure 2). No significant difference was found by species or animal age (p = 0.28 and p = 0.92, respectively), but an increasing gradient in prevalence rates was observed from north to south; the effect of latitude was significant (p<10^−6^) (Technical Appendix Table). The increasing prevalence rates moving from the north to the south might be related to higher small ruminant density in southern Mauritania (pastoral resources), which might increase between-herd transmission. Moreover, the movement of livestock between Mauritania and 2 countries to the south, Mali and Senegal (Technical Appendix Figure 2), was favorable for PPRV exchanges over the years.

Three suspected outbreaks of PPR were reported during January–March 2012 (Technical Appendix Figure 2). Both sheep and goats were affected; the animals, particularly young animals, had signs typical of acute PPR. Illness rate ranged from 11% to 17% and case-fatality rates from 39% to 58%. Clinical signs lasted 27–39 days. A total of 43 animals were sampled for virus detection, and 12 animals from 2 sites tested positive by RT-PCR. Seroprevalence rates were estimated on larger samples, including recovering animals in the 3 outbreak locations; these rates were high for all 3 sites: 61%, 70%, and 75% (n = 87, 31, and 12, respectively).

N-gene sequences were obtained from 2 sheep swab specimens collected in Trarza during the outbreak survey in early 2012 (deposited in the GenBank under accession nos. KF483658 [Mauritania1_2012] and KF483659 [Mauritania5_2012]). These isolates were placed in a phylogenetic tree built from PPRV sequences recently collected in western (Senegal, Mali) and northern Africa (Morocco), as well as isolates from other parts of the world retrieved from GenBank. Phylogenetic analysis involved 255 nt located on the C terminus end of the NP gene of the virus (84 aa). The PPRV strain from Mauritania belonged to lineage II (Figure). Sequences were close to, but distinct from, those collected in Senegal and distinct from those identified in Morocco and northern Africa (lineage IV).

Our study results highlight 2 PPRV epidemiologic systems: northern Africa, where all identified PPRVs belonged to lineage IV and were closely related to PPRV initially identified in Sudan (*5*); and western Africa, where all identified PPRVs belonged to lineages I and II (*3*,*4*). This information might be useful for the design of regional control strategies. Ongoing monitoring of PPRV in Mauritania is needed to watch for the possible spread of PPRV lineage IV from northern Africa.

Technical Appendix Small ruminant density in Mauritania, peste des petits ruminants virus seroprevalence rates in 2010, and reported peste des petits ruminants outbreaks in early 2012.
