# Supplementary material for: Peste des Petits Ruminants Virus, Mauritania
Source: Emerg Infect Dis. 2014 Feb;20(2):334–6. doi: 10.3201/eid2002.131345 (PMC3901501; doi:10.3201/eid2002.131345)
Supplement: Technical Appendix — Small ruminant density in Mauritania, peste des petits ruminants virus seroprevalence rates in 2010, and reported peste des petits ruminants outbreaks in early 2012. [file 13-1345-Techapp-s1.pdf]

Article DOI: http://dx.doi.org/10.3201/eid2002.131345

Peste des Petits Ruminants Virus, Mauritania

Technical Appendix

Technical Appendix Table. Serologic prevalence rate fitted with a beta-binomial regression model and 95% confidence interval for peste des petits ruminants in Mauritania, 2010 (n = 1,904 sheep and goats)

Latitude	Fitted prevalence rate	Lower limit	Upper limit
15.1; 16.2	0.65	0.59	0.71
16.2; 17.3	0.34	0.28	0.39
17.3; 18.4	0.21	0.14	0.29

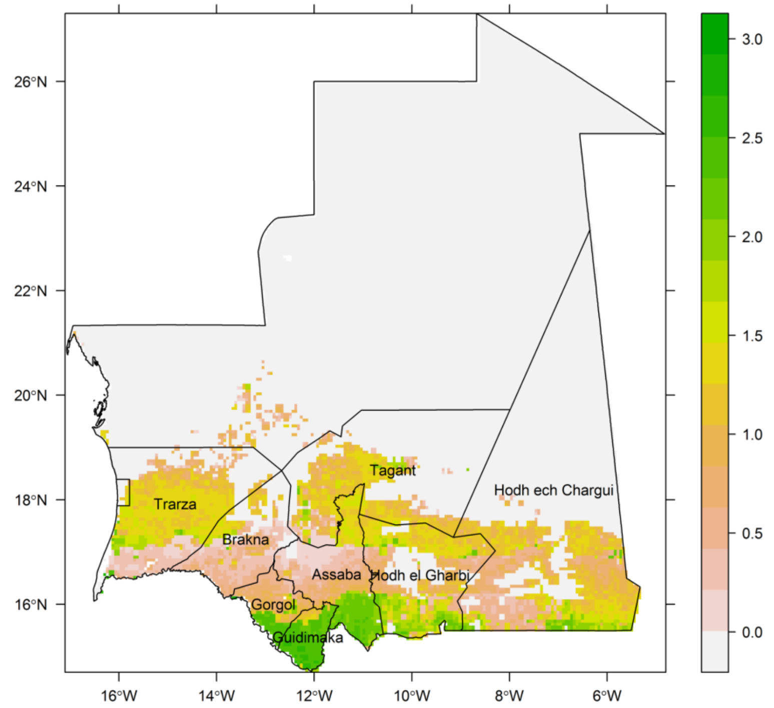

Technical Appendix Figure 1. Small ruminant density in Mauritania: number of sheep and goats by square kilometer (log_{10} scale). Densities were adjusted to match 2005 national totals (*1*).

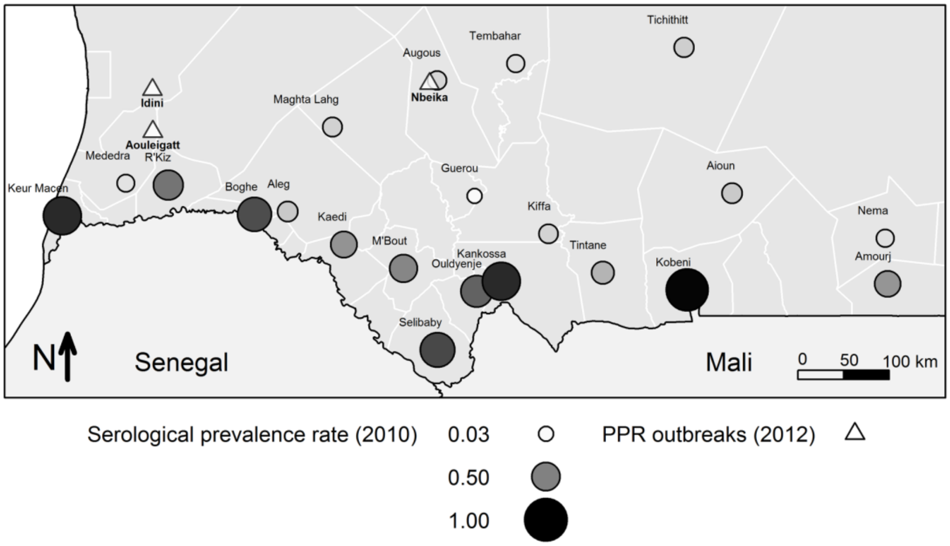

Technical Appendix Figure 2. Observed peste des petits ruminants (PPR) virus seroprevalence rates in Mauritania, 2010 (n = 1,904 sheep and goats), and reported peste des petits ruminants outbreaks in early 2012. Circle size is proportional to PPR seroprevalence rate.

Reference

1. Food and Agriculture Organization of the United Nations. Observed livestock densities. Rome: The Organization; 2007 [cited 2013 Oct 15]. http://www.fao.org/ag/againfo/resources/en/glw/GLW_dens.html
